# Association of Toll-Like Cell Receptors TLR2 (p.Arg753GLN) and TLR4 (p.Asp299GLY) Polymorphisms with Indicators of General and Local Immunity in Patients with Atopic Dermatitis

**DOI:** 10.1155/2017/8493545

**Published:** 2017-05-16

**Authors:** Yury A. Tyurin, Anton F. Shamsutdinov, Nikolay N. Kalinin, Alsou A. Sharifullina, Irina D. Reshetnikova

**Affiliations:** ^1^Department of Biochemistry and Clinical Laboratory Diagnostics, Kazan State Medical University, 49 Butlerov Street, Kazan 420012, Russia; ^2^Laboratory of Immunology and the Development of Allergens, Kazan Research Institute of Epidemiology and Microbiology, 67 Big Red Street, Kazan 420015, Russia; ^3^Institute of Fundamental Medicine and Biology, Kazan Federal University, 18 Kremlevskaya Street, Kazan 420008, Russia; ^4^The I.M. Sechenov First Moscow State Medical University, 8-2 Trubetskaya Street, Moscow 119991, Russia

## Abstract

A whole group of polymorphisms of genes involved in the formation of the epidermal barrier, immune responses, and their regulation is important in the formation of atopic phenotype. The purpose of the study is to determine the relationship of polymorphisms of genes of Toll-like receptors TLR2 and TLR4 with clinical and immunological parameters in atopic dermatitis patients in a “case-control” study. Polymorphisms of genes TLR2 (p.Arg753Gln) and TLR4 (Asp299Gly) were detected by PCR. Parameters of the state of innate and adaptive immunity were assessed by the level of local production of sIgA, cytokine profile of blood serum for IL-4, IL-10, and IFN-*γ*. Biological samples from 50 people with allergic pathology, aged 4.5 to 35 years, and 100 healthy individuals (controls) were analyzed. Observed dysregulation of cytokine production (IL-4, IL-10) in patients with heterozygous polymorphic genotypes probably reflects an imbalance of Th1/Th2/Th17 regulation of immune system response in these individuals.

## 1. Introduction

Human allergic diseases are considered today a medical issue of global significance which leads to a considerable reduction in the quality of life and public health. Extensive studies of the last two decades on the pathogenesis of allergic diseases suggest that they are associated with a number of genetic and environmental factors and also with the interaction of these factors, all of which lead to a quite complex pathogenesis and considerable difficulties for a rational therapy. Molecular and genetic studies of the last 20 years have shown that there are several hundreds of gene mutations involved in the development of an atopic phenotype [1]. In the case of atopic dermatitis, for example, there were identified significant associations with gene polymorphism regarding those genes involved in the development of the epidermal barrier, the appearance of immune responses, and their regulation [1]. Also important are the studies on associations between different polymorphic forms of genes controlling innate and adaptive immune responses in the pathogenesis of allergic diseases. Dysfunctions of the skin barrier are significant factors in the pathogenesis of atopic dermatitis, being this a matter recognized by the majority of researchers [1, 2]. Toll-like receptors (TLR) constitute one of the groups of receptors in the immune system involved in inflammatory responses of various cell types to microbial antigens [3]. It has been shown that TLR2 and TLR4 cell receptors participate in the development of innate and adaptive immune responses to lipoteichoic acids forming the cell wall of Gram-positive bacteria and also to viral proteins and LPS of Gram-negative bacteria [4]. A number of studies have shown that the clinical significance of the Toll-like receptor 2 rs5743708 (c.2258G>A) mutation (polymorphism) is characterized by the substitution of a guanine nitrogenous base with adenine in the TLR2 gene at nucleotide 2258 from the start codon (missense mutation), which leads to a change in the primary amino acid sequence at position 753 with a replacement of arginine (Arg) with glutamine (Gln). The rs4986790 (c.896A>G) mutation is another clinically significant Toll-like receptor 4 missense mutation, consisting of a nonsynonymous substitution of a nitrogenous base of adenine (A) with guanine (G). As a result, Asp is substituted with Gly at position 299 (Asp299Gly) of the primary amino acid sequence of the TLR4 protein receptor [5]. The goal of the present research (actually, a random case-control study) was to elucidate how the polymorphisms of genes TLR2 and TLR4, which encode the TLR2 and TLR4 receptors of the immune response system, are associated with clinical and immunological indicators of atopic dermatitis.

## 2. Material and Methods

### 2.1. Patients and Samples

The study considered biological samples obtained from two groups of individuals. The first group was composed of 50 individuals, comprising 25 people with atopic dermatitis and 25 with atopic dermatitis combined with either allergic rhinitis or allergic bronchial asthma. The age of patients in this group ranged from 4,5 to 35 years. In accordance with the modern classification of atopic dermatitis, the distribution of the study patients by diagnosis was as follows:

1. Atopic dermatitis, pediatric, common, moderate (SCORAD 50–89), with a high serum total IgE level (>350 IU/mL), and continuously recurrent (*n* = 15)
2. Atopic dermatitis, adolescent, common, moderate, (SCORAD 45–75), recurrent, with a moderately high total IgE level (200–350 IU/mL) (*n* = 10)
3. Atopic dermatitis, localized, moderate (SCORAD 45–70), recurrent, combined with allergic persistent rhinitis, household sensitization, and a total IgE level of >250 IU/mL (*n* = 15)
4. Atopic dermatitis, localized, moderate (SCORAD 50–65), combined with atopic bronchial asthma, persistent, mild, without signs of respiratory failure, household sensitization, and a total IgE level of >280 IU/mL (*n* = 10).

The gender ratio is 1 : 1.5 (20 male and 30 female). All patients of the study belong to the European group by race, and by ethnicity, they are Tatar (55%), Bashkir (5%), and Russian (40%) (based on the questionnaire findings).

The second group (control group) consisted of 100 persons (medical personnel) without symptoms of atopic dermatitis and aged from 21 to 39 years. The gender ratio is 1 : 1.5 including 40 men and 60 women. By race, the controls (100 persons) belong to the European group, the ethnic composition is Tatars (60%) and Russians (40%). The control and study groups selected were comparable by gender and ethnic composition.

### 2.2. Genotyping

The TLR2 (p.Arg753Gln) and TLR4 (Asp299Gly) receptor polymorphisms were determined by polymerase chain reaction (PCR) using the “SNP-express” kits with electrophoretic detection (NPO “Lytech,” Moscow, Russia) following the manufacturer's instructions. The amplification of DNA fragments was accomplished with a “Tertsik” amplifier (DNK-Tekhnologia, Russia). The amplification products were analyzed by horizontal electrophoresis in an agar gel medium, which was visualised and imaged using a transilluminator together with the image processing system “Biotest-1” (Russia). Genomic DNA was isolated from whole blood leukocytes, and buccal epithelial cells were collected with informed consent from patients during clinical and allergic investigations at Kazan Scientific Research Institute of Epidemiology and Microbiology (KSRIEM). Genomic DNA isolation and purification were accomplished by use of the “DNA-express” and “DNK-ekspresskrov-plus” kits (NPO “Lytech,” Russia).

### 2.3. Immunological Studies

The immunological studies were performed in the clinical diagnostic laboratory at KSRIEM. In order to determine the innate and adaptive immunity conditions, we studied the local production of sIgA and found the serum cytokine profile for such cytokines as IL-4, IL-10, and IFN-*γ*. Nasal secretion was collected with a cotton swab introduced in the middle nasal concha for 30 seconds, and then it was added with 0,25 ml of physiological solution, centrifuged for 10 minutes at 1200 rpm to precipitate cell components, collecting subsequently the supernatant and freezing it at −20°C. The concentrations of sIgA and cytokines in the secretion and serum samples were determined by immunoassay using the “IgА sekretornyi-IFA-BEST,” “Interleukin-4-IFA-BEST,” “Gamma-Interferon-IFA-BEST,” and “Interleukin-10-IFA-BEST” kits (ZAO “Vektor-Best,” Novosibirsk, Russia) according to the manufacturer's recommendations.

### 2.4. Statistical Analysis

The statistical analysis of the data included a test to estimate the genotype distribution deviations from the Hardy–Weinberg–Castle law using the *χ*^2^ test with Yate's correction. Subsequently, we constructed a 95% confidence interval (CI) for the mean values (M) and calculated the standard deviation of the mean values in the sample (SD). The significance of different quantitative indicators was assessed by a *t*-test for unequal variances [6].

## 3. Results

The distribution of alleles and genotypes of the *TLR2* and *TLR4* receptor polymorphisms in the tested groups was consistent with the Hardy–Weinberg–Castle law and did not deviate from the equilibrium. The distribution of genotypes in the general sample for the *TLR2* and *TLR4* receptors polymorphisms was as follows: homozygous—91,63%, heterozygous—8,37%, and homozygous for a mutant allele—0%.

The frequency distribution of alleles and genotypes of the polymorphic variants of TLR2 (*rs5743708*) and TLR4 (*rs5743708*) receptor genes in the study groups is comparable to the results of previous studies performed in the Russian Federation on the Russian population (the Chelyabinsk region) [7]. It is also consistent with the frequency distribution of alleles and genotypes of these polymorphisms in other geographic populations [8].

### 3.1. Distribution of the Frequency of Appearance of the TLR2 Receptor Gene Polymorphism rs5743708 in the Tested Groups

The frequency of the *G/G* homozygous genotype in patients with atopic dermatitis was 80,0% (40 patients), while in the control group of healthy individuals it was 94,0% (94 individuals). For the *G*/*A*^∗^ heterozygous genotype, the frequency in patients with atopic dermatitis was 20,0% (10 patients), while in the control group it was 6,0% (6 individuals); therefore, the differences were significant (*χ*^2^ = 7,4, *p* value *χ*^2^ < 0,01). Thus, we found that the *G/G* homozygous genotype was 1,17 times less frequent in the group of patients with atopic dermatitis than it was in the group of healthy individuals, whereas the *G*/*A*^∗^ (heterozygous) genotype was found to be 3,3 times more frequent in the first group than in the second.

**Figure 1:**
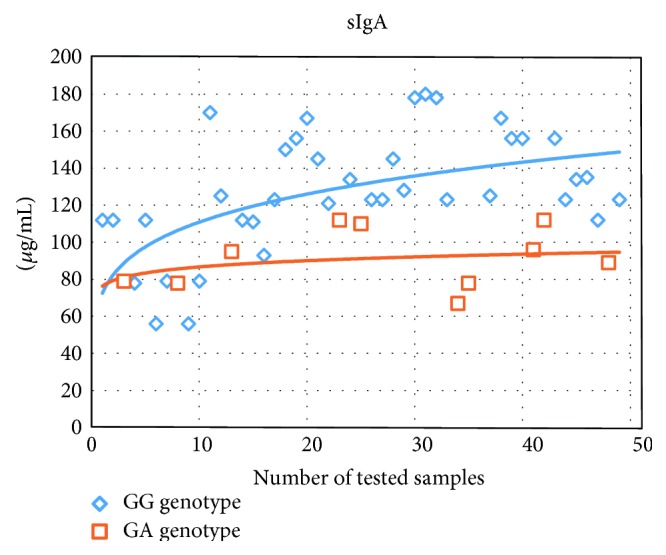
Distribution of sIgA concentration in nasal secretion in patients with atopic dermatitis with different genotypes for the TLR2 receptor polymorphism *rs5743708.*

When comparing the serum levels of cytokine *INF-γ* for patients with atopic dermatitis having different genotypes, we detected a significant reduction of cytokine *INF-γ* (more specifically, it was 1,5 times lower in the group of patients having the polymorphic (heterozygous) genotype) and also an increase in the levels of *IL-4* and *IL-10* (correspondingly, 1,4 and 1,8 times higher; see Table 1). In the group of patients having the polymorphic genotype, we found a significant reduction in the level of *sIgA* in nasal secretion, being 1,4 times lower than it was in the group with the homozygous genotype (*GG*-131 genotype: 1 *μ*g/mL and *GA* genotype: 93,5 *μ*g/mL; *p* < 0,05); see Figure 1.

The *A*^∗^/*A*^∗^ polymorphic homozygous genotype was not found in the sample of sick and healthy individuals. In general, the *TLR2* gene *G* allele frequencies were 90,0% in the group of patients with atopic dermatitis and 97,0% in the control group. Furthermore, the *A*^∗^ (rs5743708) polymorphic allele was found in 10 patients (10,0%) with atopic dermatitis and in 3 healthy individuals (3,0%) (*p* value *χ*^2^ < 0,05).

### 3.2. Distribution of Allele and Genotype Frequencies for the TLR4 Receptor Gene Polymorphism rs5743708 in the Tested Groups

In the group of patients with atopic dermatitis, the *TLR4 A/A* genotype frequency was equal to 87,0% (43 persons), while in the control group it was 91,0% (91 persons). The *A*/*G*^∗^ polymorphic heterozygous genotype was found in 7 patients with atopic dermatitis (12,0%) and also in 9 individuals from the control group (9,0%) (*p* value *χ*^2^*p* > 0, 05). We did not detect the *G*^∗^/*G*^∗^ polymorphic homozygous genotype in either group. The *TLR4* gene *А* rs4986790 allele had a frequency of 92,4% in the group of sick individuals and 94,0% in the control group; the *G*^∗^ mutant allele had frequency 3,5% in the first group and 4,5% in the second, hence revealing no significant difference (*p* value *χ^2^* > 0,05).

**Figure 2:**
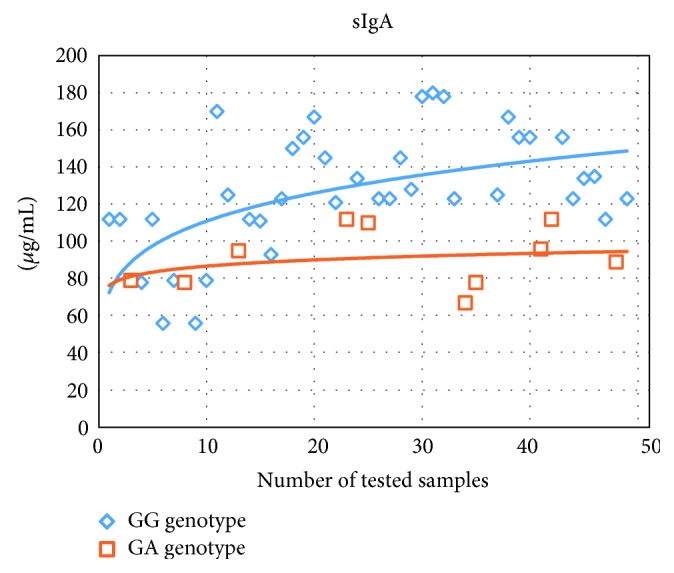
Distribution of sIgA concentration in nasal secretion in patients with atopic dermatitis with different genotypes for the TLR4 receptor polymorphism rs4986790.

By comparing the serum levels of interferon INF-*γ* in patients with atopic dermatitis, we detected a significant reduction of this cytokine level in the group of patients with polymorphic genotype (i.e., heterozygous), being 1,6 times lower than its level in the homozygous group (see Table 2). Also, we noticed a significant increase in the concentrations of serum interleukins IL-4 and IL-10 for the heterozygous group, specifically 1,3 and 1,6 times higher than the corresponding concentrations in the homozygous group (Table 2). In the group of patients having the polymorphic genotype (heterozygous group), we observed a reduction of the *sIgA* level in nasal secretion, being 1,4 times lower than that in the group of homozygous patients (АА genotype: 129,1 *μ*g/mL and AG genotype: 90,0 *μ*g/mL; *p* < 0,05); see Figure 2.

Subsequently, we compared the serum levels of cytokines and secretory immunoglobulin A in nasal secretions for the groups of patients with atopic dermatitis having different genotypes for the TLR2 and TLR4 gene polymorphisms and also for the control group (group of healthy individuals without symptoms of atopy).

We found that serum levels of interferon (INF-*γ*) in healthy individuals were higher than those in the case of patients with atopic dermatitis (see Table 3). Moreover, there were significant differences in the level of serum interferon INF-*γ* in healthy individuals compared to the corresponding level in patients with atopic dermatitis having either homozygous or heterozygous genotypes (Table 3). Additionally, we proved that serum level of IL-4 and IL-10 was 1,6 times higher but only in the groups of sick individuals with heterozygous genotypes for the gene polymorphisms considered here (compared to the corresponding levels in healthy individuals). No significant differences were detected in healthy individuals for the serum levels of cytokines depending on genotype.

When comparing serum cytokine INF-*γ* concentrations, there was a significantly decreased level of this cytokine in the atopic group. It was almost 1.4 times as high as compared to that in the control group. This pattern is also observed in the group of healthy individuals with polymorphic genotypes of the TLT-2 receptor (G/A^∗^) and TLR4 (A/G^∗^). Serum concentrations of this cytokine were as a rule higher in this group than those in patients. When studying the INF-*γ* concentrations, it was also found out that concentrations of this cytokine were even lower in the group of patients with atopic dermatitis, having polymorphic genotypes of Toll-receptors, than in the patients without a polymorphic allele in the genotype.

A comparison of the recurrence rate of atopic dermatitis over the last 3 years in subgroups of patients with a polymorphic heterozygous genotype (G/A^∗^) and a homozygous “protective” (GG) TLR2 receptor demonstrated that clinical episodes of dermatitis recurrence were significantly more often recorded (as twice as often, *p* = 0.03) in a subgroup of patients with atopic dermatitis and a mutant allele of the TLR2 gene.

## 4. Discussion and Conclusion

Toll-like receptor activation leads to the development of transcription factors in the cell cytoplasm; subsequently, these factors enter the nucleus, where they bind to promoter elements of genes involved in the expression of inflammatory mediators and cytokines, major histocompatibility complex II types, adhesins, and costimulating cells [9]. Genetic mutations in the genes encoding this type of receptors are associated with an increase in the risk of developing immune-mediated diseases such as atopic dermatitis, bronchial asthma, and allergic rhinitis, as well as autoimmune and oncological processes [10].

The *TLR2* receptor gene is located on chromosome 4. In this study, we detected the *TLR 2* gene *rs5743708* polymorphism, which is characterized by a substitution of guanine (*G*) with adenine (*A*) at nucleotide position 2257 from the start codon, this leads to a replacement of *Arg* with *Gln* at position 753 in the amino acid sequence of the receptor. The presence of this polymorphism causes a dysfunction of cell activation processes through the TLR2 signaling pathway [11].

The frequency of the *G*/*A*^∗^ polymorphic (heterozygous) genotype and, correspondingly, that of the *TLR2* gene A^∗^ (*R753Q*, rs5743708) polymorphic allele was higher in the studied group of patients with moderate atopic dermatitis than the corresponding frequencies in the group of healthy individuals. Possibly, the presence of the specified polymorphic allele is, as reported by Ahmad-Nejad, P., 2004, a predictor of a more severe course of atopic dermatitis [4]. When we determined the serum levels of interleukins in patients with the heterozygous genotype containing the *TLR2* gene *А*^∗^ (*R753Q*, *rs5743708*) polymorphic allele, we also detected quite high levels of *IL-4* and *IL-10* compared to those found in healthy individuals and patients with homozygous genotype, without the mutant allele.

The *TLR4* receptor gene is located on chromosome 9 (9q33.1). As a result of the *rs4986790* (c.896A>G) missense mutation in the *TLR4* receptor gene, a replacement of *Asp* with *Gly* at position 299 (*p.Asp299Gly*) occurs in the primary sequence of the receptor protein, leading to a disruption of both the ligand-binding and the coreceptor functions of the *TLR4* receptor, and as a consequence inducing a weak cell response to microbial antigens [12, 13]. We did not find the *G*^∗^/*G*^∗^ polymorphic homozygous genotype in neither the tested groups of patients with atopic dermatitis nor the group of healthy individuals. In the case of the *TLR4* polymorphic allele *А rs4986790*, we did not detect significant differences between the group of sick individuals and that of healthy persons.

Atopic patients with genetic polymorphisms (p.Arg753Gln, rs5743708) of TLR2 and (Asp299Gly, TLR4, and rs4986790) TLR4, as our study has demonstrated, have higher levels of Th2 cell activation cytokines (IL-4 and IL-10). Cytokine IL-10 is produced by Th2-cells and at present can be considered as an antagonist to several cytokines. In particular, it suppresses IFN-*γ* production. Moreover, it inhibits the proliferative response of T cells to allergens and mitogens and also suppresses the secretion of monocyte-activated IL-1*β*, IL-6, and TNF. At the same time, IL-10 stimulates IgE secretion and synthesis.

The study of serum levels of cytokines in our investigation showed that patients with heterozygous genotypes containing polymorphic alleles present a lower concentration of interferon (INF-*γ*) than patients without the polymorphic allele in the genotype. It is possible that the lower concentration of interferon INF-*γ* in these patients reduces the stimulatory effect of cytokine on the neutrophil (neutrocytes) system, which is likely to increase the susceptibility to viral and bacterial infections in these patients. A reduction of the activating effect of interferon *INF-γ* combined with a lower *sIgA* level in the upper respiratory tract may account for a low colonization resistance of mucous membranes against conditionally pathogenic microbiota and a low capacity of secretions to neutralize antigens and allergens of microorganisms.

The reduction of both the skin barrier function and the activity of its immune antimicrobial factors observed in patients with atopic dermatitis is caused by numerous genetic mutations affecting genes controlling the synthesis of cytokines and Toll-like receptors [2].

The decline in the stability of the skin to microorganisms, which is observed in patients with atopic dermatitis and is caused by dysfunctions of the immune regulation generated by mutations of the TLR receptors, is characterized by the development of severe and difficult-to-treat forms of dermatitis; at the same time, it leads to an increase in the flow of allergens contained in the epidermis components, ticks (mites), and microorganisms, adding complications of this disease linked to local infections [2, 3].

Cytokine INF-*γ* is actively produced by NK cells; it increases the expression of the major histocompatibility complex (MHC) class II molecules on a cell surface and enhances phagocytosis and mechanisms of innate immunity. The decreased baseline concentrations of this cytokine detected in atopic patients might be to some extent due to an imbalance in the Th1/Th2-cell system, with a prevalence of Th2-activated lymphocytes in atopy. Taking into account that this cytokine is produced by Th1 cells, their suppression in an atopic phenotype might result in a decreased baseline INF-*γ* level. Genetic polymorphisms (p.Arg753Gln, rs5743708) of TLR2 and (Asp299Gly, TLR4, and rs4986790) TLR4 genes are probably to contribute to a decrease of cell activation that further modulates a suppressor effect on Th1 cell activation and enables decreased levels of proinflammatory Th1 cytokines. Cells of the Th1 differentiation serve as suppressors of IgE response and IL-4 secretion. This activity of Th1 is mainly associated with IFN-*γ*. Therefore, any factor contributing to the Th1 differentiation by itself inhibits the development of Th2 and allergic processes. These factors also include IL-12 and IFN-*γ*.

Finally, the high degree of dysregulation detected by our study in the production of cytokines (IL-4, IL-10) in patients with polymorphic heterozygous genotypes may reflect a Th1/Th2/Th17 imbalance in the regulation of the immune system response in these individuals.

Thus, we have established that the TLR2 gene polymorphism (*p.Arg753Gln*, *rs5743708*) and the TLR4 gene polymorphism (p.Asp299Gly, rs4986790) are significant for the pathogenesis of dysregulations in the expression of cytokines by cells (immune system, skin, dendritic cells, and keratinocytes) expressing the TLR2 and TLR4 receptors, which control, differentiate, and regulate the balance of Th1/Th2/Th17 subpopulations of lymphocytes involved in immune responses in cases of atopy. Given that the TLR2 and TLR4 receptors are in a certain way cellular “sensors” able to act when exposed to exogenous stress signals and microbial antigens (pathogen-associated molecular patterns (PAMPs)), including significant allergens, we claim that the genetic variations of Toll-like receptors can notably influence the susceptibility, severity, and outcome of allergic diseases in humans.

## Figures and Tables

**Table 1:** Serum cytokine profile of patients with atopic dermatitis with different genotypes for the TLR2 receptor polymorphism rs5743708.

Cytokine profile	Genotype	
	GG (*n* = 40) “protective”	GA (*n* = 10) “polymorphic”
INF-*γ* (pg/mL)	11,69 (95% CI 11,0–11,7)	7,82 (95% CI 7,0–8,7)^∗^
IL-4 (pg/mL)	14,63 (95% CI 13,2–16,11)	20,4 (95% CI 10,8–30,3)^∗∗^
IL-10 (pg/mL)	13,2 (95% CI 11,7–14,7)	23,9 (95% CI 15,6–32,2)^∗∗^

*t*-test, differences are significant: ^∗^*p* < 0, 05, ^∗∗^*p* < 0, 001, and 95% CI.

**Table 2:** Serum cytokine profile of patients with atopic dermatitis with different genotypes for the TLR4 receptor polymorphism rs4986790.

Cytokine profile	Genotype	
	A/A (*n* = 43) “protective”	A/G^∗^ (*n* = 7) “polymorphic”
INF-*γ* (pg/mL)	12,2 (95% CI 11,7–17,9)	7,9 (95% CI 7,2–8,4)^∗^
IL-4 (pg/mL)	15,7 (95% CI 14,1–17,6)	20,4 (95% CI 10,8–30,0)^∗∗^
IL-10 (pg/mL)	13,8 (95% CI 12,1–15,3)	21,8 (95% CI 16,2–27,4)

*t-*test, differences are significant: ∗ for *p* ≤ 0, 05 and ∗∗ for *p* ≤ 0, 01.

**Table 3:** Comparative characteristics of serum cytokine profile in healthy individuals and patients with atopic dermatitis with different polymorphic genotypes.

Cytokine profile	Genotype							
	TLR2 (rs5743708)				TLR4 (rs4986790)			
	GG		GA		AA		AG	
	Atopic dermatitis, *n* = 40	Healthy, *n* = 94	Atopic dermatitis, *n* = 10	Healthy, *n* = 6	Atopic dermatitis, *n* = 43	Healthy, *n* = 91	Atopic dermatitis, *n* = 7	Healthy, *n* = 9
INF-*γ* (pg/mL)	11,67 ± 3,7	17,1 ± 3,1^∗^	7,82 ± 3,7	17,4 ± 2,9^∗^	12,2 ± 3,8	17,8 ± 2,8^∗^	7,9 ± 4,2	15,0 ± 1,5^∗^
IL-4 (pg/mL)	14,63 ± 2,2	14,4 ± 3,2	20,4 ± 3,8	13,7 ± 4,3^∗∗^	15,7 ± 4,1	14,4 ± 3,2	20,4 ± 3,1	15,1 ± 4,5^∗∗^
IL-10 (pg/mL)	13,2 ± 3,6	14,2 ± 5,4	23,9 ± 3,5	14,5 ± 3,3^∗∗^	13,8 ± 3,3	13,8 ± 3,7	21,8 ± 4,2	14,3 ± 3,5^∗∗^

M ± SD, *t*-test, differences are significant: ∗ for *p* ≤ 0, 05 and ∗∗ for *p* ≤ 0, 01.
